# Evaluation of the Diagnostic Accuracy of Comercially Available AI-CAD Solution in Mammography Screening in Mexican Women (Mammo-MX Database)

**DOI:** 10.3390/diagnostics16040517

**Published:** 2026-02-09

**Authors:** Blanca Murillo-Ortiz, Luis Carlos Padierna, Luis Fernando Parra-Sánchez, Samanta Medinilla-Orozco, Sergio Meza-Chavolla, Samuel Rivera-Rivera, Aura Rubiela Espejo-Fonseca

**Affiliations:** 1 Unidad de Investigación en Epidemiologia Clínica, OOAD Guanajuato, Instituto Mexicano del Seguro Social, León 37328, Mexico; samamedex@hotmail.com; 2 División de Ciencias e Ingenierías, Universidad de Guanajuato, León 37150, Mexico; 3 Unidad de Detección y Diagnóstico Clínica de Mama, Instituto Mexicano del Seguro Social, Guadalajara 44300, Mexico; sergio.mezac@imss.gob.mx; 4 Coordinación de Atención Oncológica, División de Atención Oncológica en Adultos, Instituto Mexicano del Seguro Social, Ciudad de México 030020, Mexico; 5 Biopsy Unit, Salud Digna, Avenida Universidad 1338, Coyoacán, México City 04100, Mexico

**Keywords:** mammography, breast cancer, breast density, artificial intelligence

## Abstract

**Background/Objectives**: The objective of this study was to evaluate the performance of Breast-SlimView^®^, a deep convolutional neural network for the automatic classification of BI-RADS and breast density in MLO (mediolateral oblique) and CC (craniocaudal) views. **Methods**: A total of 9560 mammographic images from 2390 Mexican women (age: 54.14 ± 8.72 years) were labeled according to ACR (American College of Radiology) density (A-D) and BI-RADS 1, 2, and 3 (low risk), and BI-RADS 4 and 5 (high risk). All mammograms in the test dataset were blinded and read by two radiologists, and the consensus was taken as the reference standard. The accuracy, sensitivity, and specificity of the automated AI-based classification system was evaluated against the consensus reached by expert radiologists. **Results**: The classification of MLO and CC projections had a mean sensitivity of 0.81 (95% CI: 0.797–0.829), a specificity of 0.70 (95% CI: 0.686–0.722), and an accuracy of 0.71 (95% CI: 0.698–0.734) in differentiating between low and high risk. Good agreement was observed with ACR breast density classifications A, B, C, and D. Agreement between AI and human readers was “substantial” (Pearson’s chi-square, *p* = 0.001). **Conclusions**: AI enables accurate, standardized, observer independent classification.

## 1. Introduction

Breast cancer is one of the most prevalent types of cancer and the second-leading cause of cancer related death in female worldwide. Screening of breast cancer usually begins with the use of imaging examination, with mammography as the most common first choice of early detection, starting between ages 40 to 50 [1].

Mammograms provide images of the breasts that allow radiologists to identify suspicious or abnormal areas and classify them according to scales of probability of benignity or malignancy, for example, the Breast Imaging Reporting and Data System (BI-RADS) of the American College of Radiology (ACR) [2]. According to the latest statistics, there are approximately 20.6 million women between the ages of 40 and 69 in Mexico. However, only 20.2% underwent a mammogram in the previous year, and of those, only 85.6% received the results [3].

Mammography is a widely available screening test in many countries, and its main benefit is a reduction in breast cancer mortality of between 24% and 48% [4]. Although it offers tangible benefits, mammography is not perfect, as this diagnostic method carries a risk of over diagnosis, leading to overtreatment and anxiety in patients. In clinical practice, the risk of a false positive is approximately 20%, and the risk of undergoing a biopsy due to a false positive is 3% of all women who undergo a biennial screening between the ages of 50 and 69 [5].

In addition, the growing number of people having access to public and private healthcare generates a substantial workload for healthcare personnel responsible for the entire mammography process, especially radiologists. This whole situation has led to the need for tools able to facilitate routine diagnosis. Deep Learning-based Computer Aided Detection (CADe) and Diagnosis (CADx) systems have been adopted in medical practice, intended to help radiologists during image interpretation [6].

Most recent Deep Learning-based CAD systems use images, either alone or combined, and output a score that allows the identification of suspicious findings and the estimation likelihood of malignancy, aiming to improve the overall mammography interpretive accuracy in screening programs [7,8]. The use of artificial intelligence (AI) based on CAD algorithms improve significantly diagnostic performance, increasing the area under the ROC curve (AUC-ROC) (BSR’s: 0.813 to 0.884, GRs: 0.683 to 0.833) and sensitivity without affecting the specificity. Moreover, AI enhanced the average reading time among BSRs from 82.73 s without AI assistance to 73.04 s with AI assistance [9].

The issue of breast density is central to the discussion on artificial intelligence applied to mammography, as it constitutes an independent risk factor for breast cancer and, at the same time, one of the main sources of diagnostic variability. Taylor et al. highlight that AI-based density assessment not only improves consistency in classification but can also be integrated into broader risk stratification algorithms. This opens up the possibility of personalizing screening according to each patient’s individual profile. However, the authors caution that for density to become a clinically useful parameter in automated models, both the measurement methodology and the categorization thresholds used in different screening programs need to be standardized [10]. On the other hand, the diagnostic image quality of an examination has a significant impact on cancer detectability: inadequate positioning, image artifacts, or insufficient breast compression can reduce breast cancer detection sensitivity from 84.0% to 66.3%. Although the criteria are well defined, the subjectivity of human evaluation can lead to low reliability of scoring due to variability among readers. Recent studies have demonstrated the feasibility of AI for automatically evaluating the image quality of mammograms, with an accuracy above 85% for key quality indicators and showed near-perfect agreement with radiologists. Implementing AI-based tools may support standardized quality control and reduce workload [11].

The mean glandular dose (MGD) is an estimate of the average absorbed dose to the glandular tissues of a breast during mammography. It is measured in Gray (Gy). The most commonly accepted method of calculating the mean glandular dose is described by Dance et al. (2000) [12] MGD = Kgcs. The MGD scan provides an indication of the radiation risk to the breast during exposure. Regional and national radiology safety guidelines will use the MGD as a measure to determine diagnostic reference levels [12]. To enable quality control and comparisons between different equipment, the MGD is calculated to the standard breast which is defined as a 4.2 cm thick ACR phantom. It is a legal requirement that the MGD attached to the ACR phantom must not exceed 3 mGy. Typical MGDs are lower than this limit [13].

Breast-SlimView^®^ by HERA-MI provides information to the radiologist for decision making through specific analysis of positioning, compression, breast density, and the radiation dose received in each of the projections. Mammograms are identified as perfect, adequate, moderate, or inadequate.

Identified mammograms with appropriate breast compression and positioning are rated either perfect, good, moderate, inadequate. This study was designed to evaluate the concordance between radiologists and the Breast-SlimView^®^ system, hereafter referred to as the algorithm, in mammographic screenings in Mexican women (Mammo-MX database).

## 2. Materials and Methods

In this work, we focus on the problem of binary classification of breast cancer using mammography in the context of data with heterogeneous annotations. We have a sufficient number of well-annotated samples with ground truth confirmed by a reference standard. The Mammo-MX database (details on how to access this dataset are provided in the Data Availability section) is the first mammography dataset specifically focused on Mexican patients. It aims to advance breast cancer detection through artificial intelligence and is designed to facilitate the development, training, and validation of machine learning models as auxiliary diagnostic tools for the early identification of breast cancer.

The available samples also have labels for other classes, BI-RADS scores indicating the probability of cancer (i.e., ACR classification), breast density and viewing angle. We have access to a dataset with images labeled as BIRADS 4 and 5, with confirmed breast cancer classification. The mammography database presented in this study comprises a collection of mammograms acquired between 2023 and 2024 were obtained using a HOLOGIC^®^ Selenia Dimensions digital mammography device, at the Unidad de Detección y Diagnóstico Clínica de Mama, Instituto Mexicano del Seguro Social, which produces high-quality mammograms essential for accurate diagnostic interpretation. BI-RADS classifications were determined by experienced radiologists specializing in mammary gland interpretation.

Mammograms were classified according to the Breast Imaging Reporting and Data System (BI-RADS) density categories, as defined in the 5th edition (2013) by the American College of Radiology (ACR) [14]. The classification comprises four descriptive categories with corresponding quantitative percentage quartiles of the amount of fibro glandular tissue: A: Fatty (<25% fibro-glandular tissue), B: Scattered fibro-glandular densities (25–50%), C: Heterogeneously dense (51–75%), and D: Extremely dense (>75%).

In this study, in agreement between radiologists and the algorithm, a collaboration with AI (Breast-SlimView^®^ version 1.9.0) was carried out to promote the use of AI algorithms focused on the interpretation of screening mammograms. A total of 2390 women in the dataset were evaluated using the AI software (Breast-SlimView^®^ version 1.9.0). Importantly, no training or fine-tuning was performed on these cases prior to testing.

## 3. Results

**Figure 1:**
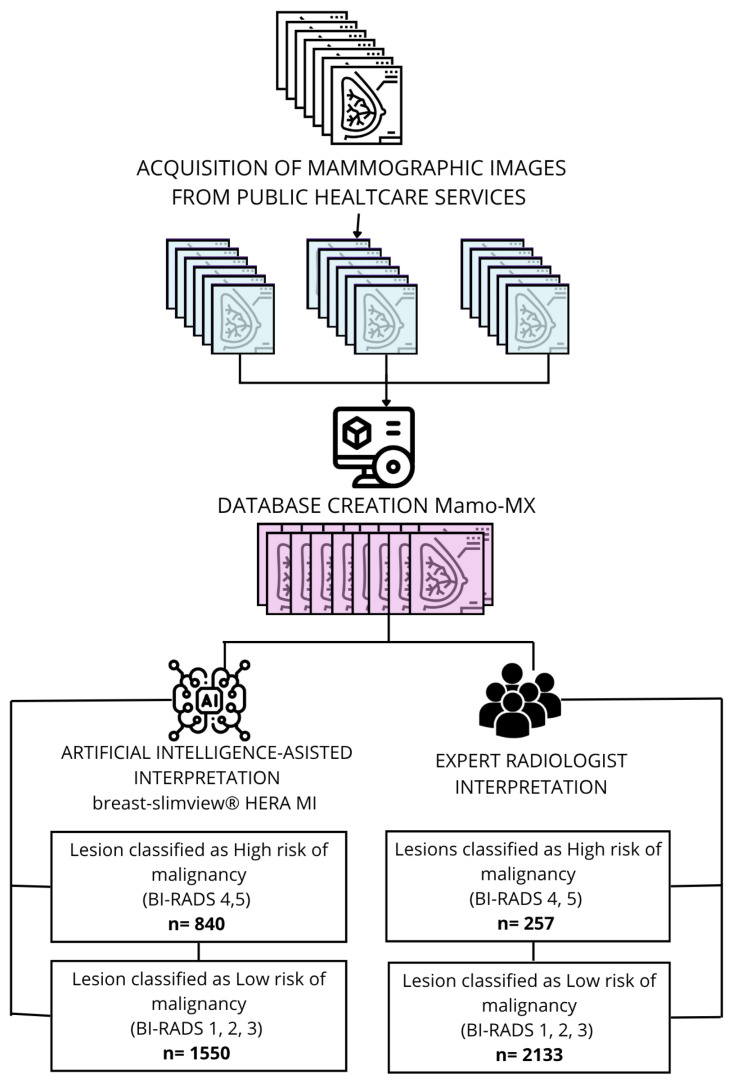
Workflow diagram and distribution of interpretation by radiologists and classification by Breast-SlimView^®^. Among the mammograms labeled as high risk by radiologists, 92 correspond to BI-RADS 4A, 100 to BI-RADS 4B, 19 to BI-RADS 4C, and 46 to BI-RADS 5. Regarding the cases classified as low risk, 856 were categorized as BI-RADS 1, 1266 as BI-RADS 2, and 11 as BI-RADS 3.

A total of 9560 screening mammograms from 2390 Mexican women were analyzed. The participants ranged in aged from 20 to 94 years, with a mean age of 54.14 ± 8.72 years. Although the AI algorithm is capable of classifying each image individually, in this study, all four projections per patient were considered, and the final BI-RADS category was assigned according to the highest rating among them. In cases where the two radiologists disagreed, the categorization provided by the more experienced radiologist was used. The algorithm, however, provides a categorical classification based on malignancy risk into three levels: low, medium, and high. For the purpose of this analysis, medium and high-risk predictions were grouped as high risk, while the low-risk category was retained as such. The workflow diagram in Figure 1 shows the distribution of cases classified per woman as having a high risk of malignancy corresponding to BI-RADS 4 and 5, and those with a low risk of malignancy corresponding to BI-RADS 1, 2, and 3, as interpreted by radiologists and classified by Breast-SlimView^®^. The number of cases classified as high risk for breast cancer by radiologists was 257, and 840 by AI. Those classified as low risk were 2133 by radiologists and 1550 by AI. Figure 1.

**Figure 2:**
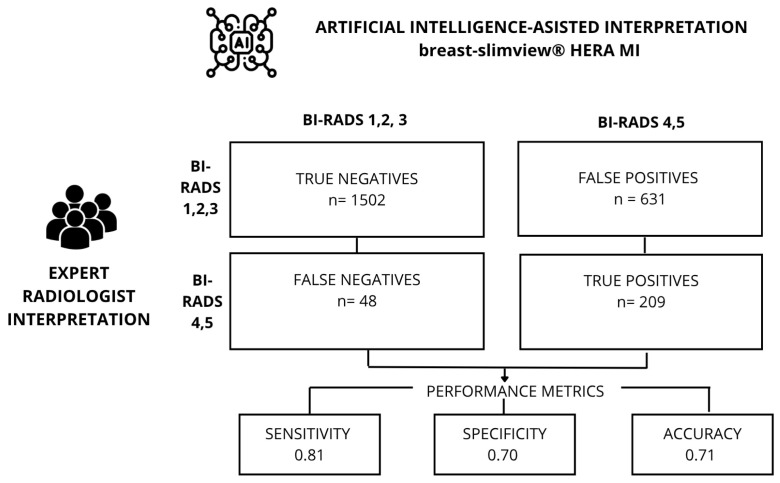
Confusion matrix and performance metrics.

Using a confusion matrix, we calculated metrics based on positive and negative predictive values. To ensure the accuracy and reliability of our results, it was essential to use metrics that allowed us to understand the performance of these algorithms, and thus, validate them. Similarly, we seek to find the best hyperparameters to optimize the algorithm’s performance. Accuracy is defined as the proportion of correct predictions (both true positives and true negatives) within the total number of cases examined. Figure 2. The Matthews correlation coefficient (*MCC*) is a statistical measure that yields a high score only if the prediction obtained good results in the four categories of the confusion matrix: true positives, false negatives, true negatives, and false positives [15]:

(1) $$MCC=\frac{c\times s-\sum_{k}p_{k}\times t_{k}}{\sqrt{\left(c^{2}-\sum_{K}p_{k}^{2}\right)\left(c^{2}-\sum_{k}t_{k}^{2}\right)}}$$

where *c* is the total number of samples, *s* is the total number of correct predictions, i.e., the sum of the diagonal in the corresponding confusion matrix; $p_{k}$ is the number of times class *k* was predicted, and $t_{k}$ is the number of times class *k* actually occurred.

The accuracy of the algorithm for breast cancer detection was evaluated by analyzing the agreement between the classification provided by expert radiologists and those generated by the AI system. An accuracy of 0.71 (95% CI: 0.698–0.734) was obtained, with a sensitivity of 0.81 (95% CI: 0.797–0.829), and a specificity of 0.70 (95% CI: 0.686–0.722).

Radiologists categorized 257 studies as highly suggestive of malignancy, and biopsies were performed on 177 of these cases to confirm the diagnosis. Among them, 124 were positive for breast carcinoma while 53 were negative for malignancy. Of the confirmed tumors, 79.03% were invasive ductal carcinoma (IDC), 18.54% were invasive lobular carcinoma (ILC), and only 2.41% were other types.

A total of 840 cases were classified by the algorithm as BI-RADS 4 and 5, and the biopsy results were confirmed in 124 women diagnosed with breast cancer. It is not feasible to determine whether these are false negatives, since radiologists did not classify 631 cases as Birads 4 and 5. There were 53 cases with negative biopsies where the algorithm classified them as BI-RADS 1, 2, and 3, which were true negatives. We propose three possible explanations for the observed discrepancies between radiologists and IA classifications. First, commercial CAD systems typically configure their classification thresholds to prioritize sensitivity over specificity, aiming to minimize false negatives in clinical screening scenarios. Second, although double reading protocols are implemented, inter-reader variability is well-documented in mammographic interpretation. As a result, some suspicious cases may still be missed or classified differently, even with this safeguard in place. Third, we acknowledge that the training data used for commercial AI systems may influence their decision boundaries. Given the relative scarcity of publicly available datasets containing confirmed cancer cases, there is a possibility that the algorithm’s learned patterns may introduce certain biases in risk stratification, potentially affecting the threshold at which cases are classified as high-risk.

**Figure 3:**
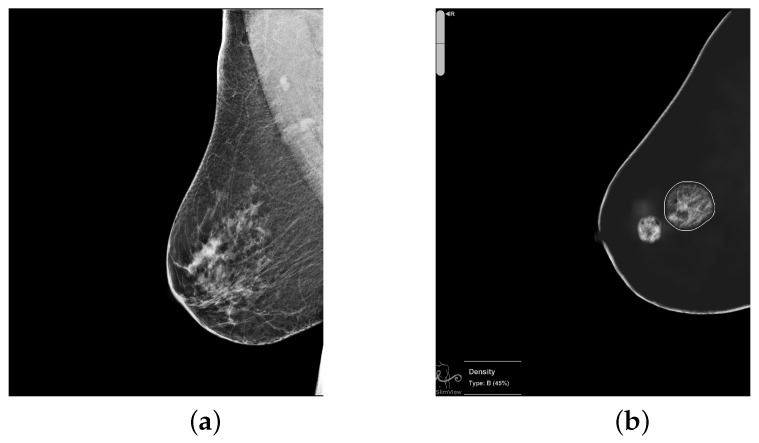
Example of disagreement between the algorithm and radiologists. The algorithm classified the case as high-risk, whereas the radiologists labeled it as low-risk (BI-RADS2). (**a**) Mammographic study categorized as BI-RADS 2 by radiologists. (**b**) Region detected by the algorithm that led to the high-risk classification.

To illustrate the discrepancies between the AI algorithm and radiologists, Figure 3 presents a case classified by the algorithm as high-risk and by radiologists as low-risk. In the image on the left, the study was categorized by radiologists as BI-RADS 2, while the image on the right shows the finding detected by the software, which led to its classification as high-risk.

### 3.1. The BI-RADS Density Classification

The percentage distribution on BI-RADS categories reported by expert radiologist against Breast-SlimView^®^ is presented in Table 1 and Table 2 for the density of the left and right breasts, respectively. A total of 2182 exams (four mammograms per exam) were analyzed, and chi-square testing revealed a statistically significant relationship (*p* = 0.001). Breast-level analysis was performed, which means that the density classification was independently assessed for each breast. A total of 208 exams were not interpreted by radiologists because these were incomplete and, therefore, were excluded. Breast density B and C was the most frequent for both breasts.

According to the interpretation of radiologists, the patterns had the following percentages within each BI-RADS A–D category. Left breast density: 20.3%, 58.3%, 19.1%, and 2.0%, respectively. Right breast density: 20.7%, 58.2%, 19.1% and 2.0%. A high degree of agreement was observed with that found by AI; the left breast was 17.47%, 41.29%, 13.27%, and 1.27%, and the right breast density was 16.1%, 42.3%, 13.8%, and 0.8%. A significant association between radiologists’ and IA classifications for both breasts was obtained using the chi-square test (*p* < 0.001). The MCC values calculated from Table 1 and Table 2 for left and right breast densities were 0.432 (95% CI: 0.399–0.465) and 0.455 (95% CI: 0.421–0.487), respectively, indicating a moderate performance with room for improvement, especially in class D. The Cohen’s kappa coefficients for the left and right breast classification were 0.469 (95% CI: 0.438–0.503) and 0.454 (95% CI: 0.420–0.487), respectively, confirming a consistent level of agreement between the radiologists’ and AI assessments.

### 3.2. Evaluation of Mammograms

A total of 9560 mammograms were included in this study. The most frequently used scale for evaluating image quality was the visualization of posterior breast tissue in craniocaudal (CC) and medio-lateral oblique (MLO) views, followed by the pectoral muscle volume determined in the MLO view. In general, positioning, artifacts, and compression were the main reasons for discarding mammograms.

The algorithm identified mammograms with appropriate breast compression and positioning: perfect in 8 cases (0.33%), adequate in 659 cases (27.57%), moderate in 1700 cases (71.12%), and inadequate in 23 cases (0.96%).

The mean age of patients was 54.14 ± 8.72 (20–94 years). The mean compression (kPa) for each projection was: craniocaudal right (CCR): 9.12 ± 4.03, medio-lateral oblique right (MLOR): 10.07 ± 3.92, craniocaudal left (CCL): 8.57 ± 3.81, medio-lateral oblique left (MLOL): 10.28 ± 3.99.

## 4. Discussion

Contemporary approaches to artificial intelligence (AI) based on deep learning have generated interest in the application of AI for breast cancer screening (BCS). The U.S. Food and Drug Administration (FDA) has approved several next-generation AI products with an indication for BCS in recent years. However, concerns regarding AI’s accuracy, appropriate use, and clinical utility persist [16].

In a systematic review of AI studies in radiology published from 2015 to 2019, five hundred and thirty-five articles were included for analysis. A total of one hundred and fifty-six (29%) studies employed customized deep learning architectures. UNet was the most popular established architecture used in 76 (14%), followed by ResNet. Ensemble methods were described in 19 (4%) cases. Where the model was previously described (313), it was modified in 275 (88%) cases and used “off the shelf” in the remainder. Supervised learning was used in 473 (88%) studies, unsupervised learning in 13, a combination of both in 8, and semi-supervised learning in 6. The methods were unclear in 38 (7%) cases. Transfer learning was not used in 284 (53%), was used in 247 (46%), and was unclear in the remainder [17].

There are a large number of studies showing that AI algorithms perform well in detecting cancer in screening mammograms. The algorithms have been trained primarily using homogeneous internal databases or those from a single institution, although there are also training programs using larger, heterogeneous, or representative databases, including data from the United Kingdom and the United States, comparing the performance of AI with that of radiologists. McKinney, S.M et al., observed an absolute reduction of 5.7% and 1.2% in false positives and 9.4% and 2.7% in false negatives (US and UK databases, respectively). The AI algorithm performed significantly better than all human readers in the reading study [18].

In the present study, the AI algorithm demonstrated its performance on a dataset of 2390 Mexican women, achieving a sensitivity of 81% and a specificity of 70%, while in a previous study reported by Tardy M and Mateus D using the same AI system on the INbreast database they obtained a sensitivity of 80.00% and a specificity of 49.03% when fixing the operating point at a malignancy probability of p>0.5 [19]. They also used Breast-SlimView^®^ to achieve a binary classification performance of AUC-ROC = 80.46 in a private dataset, and AUC-ROC = 85.23 in the INbreast dataset. The differences observed in performance with different databases reflect the importance of building large databases with different populations for training and validating algorithms. In this case, the AI algorithm used a private multivendor dataset composed of 2520 Full Field Digital Mammography (FFDM) images from four different vendors, namely, Fujifilm, GE, Hologic, and Planmed, for its training and validation. It contains 1271 benign and 1249 malignant mammograms [19].

In a study evaluating the use of a commercial AI product by 24 radiologists who retrospectively read an enriched dataset of 260 digital breast tomosynthesis cases, Conant et al. compared the independent performance of the AI system with that of radiologists who read without the AI system [20]. The mean sensitivity and specificity of the readers were 77.0% (range: 38.5–93.8%) and 62.7% (range: 22.1–84.6%), while the corresponding metrics for the AI system were 91% and 41%. In the present study, when analyzing our metrics using the algorithm, a sensitivity of 81% and a specificity of 70% were obtained.

AI can not only reduce the workload of physicians, but also continuously improve the accuracy and sensitivity of breast cancer diagnosis and treatment. Between 70% and 90% of patients with suspected breast cancer detected by mammography were ultimately diagnosed with a “false positive” [21]. In the present study, radiologists classified 257 studies as highly suggestive of malignancy, and biopsies were performed on 177 to confirm the diagnosis; 124 were positive for breast carcinoma and 53 were negative for malignancy. AI classified 840 cases as highly suggestive of malignancy, resulting in 209 true positives and 631 false positives. Without being able to perform a biopsy in these cases, it is not feasible to know if they are truly false positives. These patients need to be followed up to determine if they will develop a lesion in the next annual or biannual study.

Kunal C Potnis et al., analyzed the evaluation of FDA device regulation and future recommendations, which included nine AI products indicated for the identification of suspicious lesions in breast-conserving surgery (BCS) and the classification of mammograms. Six products used multicenter designs. Enriched data were used for eight devices, and four devices lacked details on whether the products had been externally validated. Test performance measures, including sensitivity, specificity, and area under the curve, were the main outcomes reported. Most devices used tissue biopsy as the reference standard for assessing the accuracy of BCS. Other measures of clinical utility, such as cancer stage at the time of detection, detection of interval cancers, or other outcomes, were not reported for any of the devices [16].

Lehman et al. emphasize that the main advantage of using a deep model to assess density is the significant reduction in interobserver variability, a common problem even among experienced radiologists. The acceptance of the binary output “dense/non-dense” demonstrates that AI can bring clarity and uniformity to reports, which is critical in contexts where density directly influences the indication for complementary studies, such as magnetic resonance imaging or ultrasound. The discussion emphasizes, however, that the challenge of validating this standardization across multiple centers and different populations remains, in order to ensure that the clinical utility transcends the environment in which the model was developed [22].

Winkel et al. conducted a retrospective case–control study included 122 cases and 262 age- and time matched controls (765 breasts) based on a 2007 screening cohort of 14,736 women with negative screening mammograms from Bispebjerg Hospital, Copenhagen. Digitized randomized film-based mammograms were classified independently by two readers according to two radiological visual classifications (BI-RADS and Tabár) and an interactive computerized threshold technique measuring area-based percent mammographic density (denoted PMD). Consistency was highest for low risk patterns with the following agreement within each A-D BI-RADS category: 94%, 72%, 62%, and 69%, respectively. Two-grade disagreement was only seen in one case (B/D) corresponding to 0.1% (breast based). R1 judged systematically one category higher regarding 157 of the 765 disagreed breast mammograms (21%), and only 2% were judged in a lower category compared with R2 [23].

When analyzing the distribution of breast density categories reported by radiologists in Mexican women, the percentages for each category A-D were as follows: for the right breast, 20.7%, 58.2%, 19.1%, and 2%; for the left breast, 20.3%, 58.3%, 19.1%, and 2.0%. We performed a concordance test that showed statistically significant concordance with the breast density reported by the algorithm. This shows us that we can obtain these parameters quickly and safely, identifying cases of higher risk due to increased breast density. We also explored the Matthews correlation coefficient (MCC) for its better fitness to the unbalanced-dataset scenario. The MCC values calculated for 157 densities of the left and right breast were 0.432 and 0.455, respectively; indicating a moderate performance with room 158 for improvement, especially in class D.

Other highly relevant applications of artificial intelligence besides risk classification include identifying breast density distribution and image quality. The use of AI in breast imaging offers a great opportunity to improve the quality of breast images provided to patients. Knowing the compression applied and the amount of radiation allows us to make decisions when requesting new images, such as enlargements, cones, etc. Radiology technicians play a critical role in obtaining high-quality mammographic images, not only through quality control, but also through proper compression and positioning of the breasts [24,25].

Brahim et al. demonstrated that an AI algorithm could evaluate breast position in mammograms to detect common problems that can lead to inadequate positioning, such as nipple profile, breast rotation, pectoral muscle visualization, inframammary fold, and nipple pectoral line. The algorithm was highly accurate in identifying these deficiencies, making it an excellent tool for quality control in radiology services. In our study, the algorithm identified mammograms with appropriate breast compression and positioning, perfect 8 (0.33%), adequate 659 (27.57%), moderate 1700 (71.12%), inadequate 23 (0.96%) [26].

Serwan et al. conclude that, in addition to comparing current protocols, key emerging concepts include the rationale for standardization, the benefits of improved diagnostic outcomes and reduced pain with negligible change in image quality and average glandular dose (AGD), and the recommendation for a standardization protocol of approximately 10 kPa pressure [27]. In our study, the mean compression (kPa) was as follows for each projection: craniocaudal right (CCR): 9.12±4.03, medio-lateral oblique right (MLOR): 10.07±3.92, craniocaudal left (CCL): 8.57±3.81, medio-lateral oblique left (MLOL): 10.28±3.99. Determining quality helps us improve technical conditions.

Although the algorithm achieved satisfactory results even with a dataset entirely independent from its training data, certain limitations must be acknowledged. The study population was specific, with all images acquired using the same mammography unit, which may restrict generalizability. Furthermore, only a limited number of cases had biopsy-confirmed diagnoses, which constrains the ability to definitively identify false-positive or false-negative cases. Finally, as this AI model does not provide access to its internal probability outputs, we were unable to perform a more detailed evaluation using alternative thresholds or advanced performance metrics.

## 5. Conclusions

This study underscores the potential of machine learning methods to enhance mammogram interpretation and breast density assessment. The evaluated AI algorithm demonstrated moderate level of agreement with the radiologists’ classifications (according to MCC, kappa, specificity, and sensitivity metrics) in mammograms of Mexican women. This finding is particularly relevant considering that the AI system had not been trained with cases from this populations.

Technical evaluation remains essential for ensuring quality control and optimizing image interpretation. Relevant factors influencing image quality such as radiation dose, compression, and breast positioning were accurately quantified by the AI system, providing complementary information for clinical assessment.

Although the clinical utility of current AI systems in breast cancer screening continues to be a matter of investigation, the results of this study provide evidence on their performance and generalization capabilities when applied to a population different from the one used during training. 

## Figures and Tables

**Table 1:** Cross-tabulation based on left breast density.

	Radiologists				
The Algorithm	A	B	C	D	Total
**A**	320	197	3	0	520
**B**	151	927	146	5	1229
**C**	4	163	207	21	395
**D**	1	8	23	6	38
**Total**	476	1295	379	32	2182

**Table 2:** Cross-tabulation based on right breast density.

	Radiologists				
The Algorithm	A	B	C	D	Total
**A**	315	166	3	0	484
**B**	158	960	145	2	1265
**C**	2	159	220	29	410
**D**	0	6	15	2	20
**Total**	475	1291	383	33	2182

## Data Availability

The Mammo-MX dataset is available in a public repository and as a data paper which can be accessed at https://zenodo.org/records/17740027 (accessed on 23 December 2025). Interested researchers can also request the dataset directly to the corresponding author.
